# Survey of the Synanthropic Flies Associated with Human Habitations in Ubon Ratchathani Province of Northeast Thailand

**DOI:** 10.1155/2012/613132

**Published:** 2012-08-09

**Authors:** Tarinee Chaiwong, Thanyakarn Srivoramas, Kom Sukontason, Michelle R. Sanford, Kittikhun Moophayak, Kabkaew L. Sukontason

**Affiliations:** ^1^College of Medicine and Public Health, Ubon Ratchathani University, Warinchamrap, Ubon Ratchathani 34190, Thailand; ^2^Faculty of Medicine, Chiang Mai University, Muang, Chiang Mai 50200, Thailand; ^3^Pathology, Microbiology and Immunology, University of California, Davis, CA 95616, USA; ^4^Bung Borapet Research and Training Center, Mahidol University Nakhonsawan Campus, Nakhonsawan 60000, Thailand

## Abstract

Synanthropic fly surveys were performed to determine the species composition and abundance in Ubon Ratchathani province in Northeast Thailand. Adult fly collections were conducted in various human habitations from two districts—Muang Ubon Ratchathani and Warinchamrap, at fresh-food markets, garbage piles, restaurants, school cafeterias, and rice paddy fields. Customized reconstructable funnel fly traps baited with 250 g of 1-day tainted beef were used for fly collections from September 2010–February 2011. A total of 3,262 flies were captured, primarily consisting of three families including: Calliphoridae (6 species), Muscidae (3 species), and Sarcophagidae (11 species). The blow fly, *Chrysomya megacephala*, and the house fly, *Musca domestica*, were the dominant species collected from both districts at all collection sites. *C. megacephala* was predominant in paddy fields, restaurants and garbage piles, while *M. domestica* was numerically dominant in fresh-food markets and school cafeterias. The current survey identified various species of synanthropic flies with close associations to humans and with the ability to transmit human pathogens in Ubon Ratchathani province; providing crucial information that may be used for developing control and sanitation management plans in this particular area.

## 1. Introduction

Synanthropic flies are those flies which are adapted to live in close association with human habitations and are capable of transmitting human pathogens either mechanically or biologically through this close relationship [1]. The link between human pathogens and fly transmission is due to the fact that adults feed on animal manure, trash, human excrement, and other decaying materials; readily moving between these habitats and food, food preparation surfaces, and humans themselves [2]. Species of flies in the families Muscidae (house flies, latrine flies, and relatives), the Calliphoridae (blow flies and bottle flies), and the Sarcophagidae (flesh flies) have evolved to live in close association with human development. There are over 50 species of synanthropic flies that have been reported to be associated with unsanitary conditions and involved in the dissemination of human enteropathogens in the environment [2]. In addition, the larvae of these flies can also cause myiasis in human and livestock [3]. The annoyance and public health risks associated with large populations of such flies is thus considerable.

Population densities of synanthropic flies are largely tied to sanitation practices such that they are abundant in both urban and rural areas where unsanitary conditions exist and are usually scarce when sanitary conditions are enforced [4]. Synanthropic fly population surveys are often conducted with respect to enteropathogenic bacteria transmission [5–8] as well as some gastrointestinal parasites [9]. Another notable feature of synanthropic fly populations is their propensity for very rapid fluctuations in population density, often with adult numbers increasing by up to two orders of magnitude in a few days [10]. Thus, survey data that is collected over long periods of time is required to capture the variation present in fly population densities, species composition, and richness.

Synanthropic fly surveys have been conducted in many countries of the Southeast Asian region. In Malaysia, the predominant species recovered from surveys was *M. domestica*, but* Sarcophaga* spp. were not identified in these studies [11, 12]. In Thailand, surveys of Muscidae and Calliphoridae have shown that *M*. *domestica,* and *C. megacephala,* are the two most prevalent species collected [13–16]. However, to date, no survey of the Sarcophagidae in Thailand has been made. Sarcophagidae are technically challenging to identify and require examination of the male genitalia. Therefore, they are often not resolved to specific taxonomic resolution.

There is a great need for long-term surveys in Thailand of the three major synanthropic fly families to help determine the effectiveness of sanitation practices, identify breeding sites, determine fly population fluctuations, determine the need for control measures, and identify other insect species that will inadvertently be affected by the chosen control methods [12]. The broad aim of this study was to determine the synanthropic fly species composition in two areas of Ubon Ratchathani province in Northeast Thailand: Muang Ubon Ratchathani and Warinchamrap districts, and to examine population fluctuations, species composition, and abundance over a six-month study period. Baseline synanthropic fly data will provide a means to generate integrated pest management plans for fly control and determine the impact on the human community.

## 2. Materials and Methods

### 2.1. Collection

**Figure 1:**
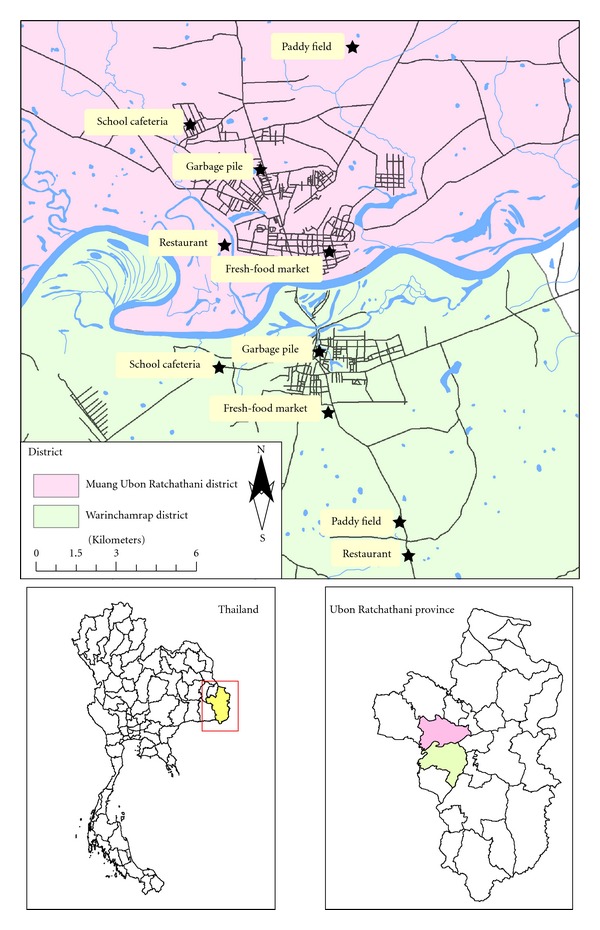
Ten collection sites for adult fly collections in Muang Ubon Ratchathani and Warinchamrap districts, Ubon Ratchathani province Northeast Thailand.

Fly surveys were conducted in two areas of Ubon Ratchathani province in Northeast Thailand—Muang Ubon Ratchathani district (the capital district of the province) and Warinchamrap district. Collections were carried out at ten collection sites in each of the two districts during September 2010–February 2011. The collection sites in each district were selected to include places where people and flies would most likely interact with respect to enteropathogenic bacterial transmission, including fresh-food markets, garbage piles, restaurants, school cafeterias, and rice paddy fields where flies have the opportunity to feed on both waste or unsanitary materials and human food items. The locality of each study area was georeferenced by a Garmin GPSMP 60CSx (Figure 1).

Adult fly collections were performed using individually deployed traps separated by approximately 1 km at each site. Customized funnel trap kits, designed by K. Sukontason [16], were deployed at each collection site in the morning (09:00–12:00). This trap consists of three components: (1) a polyvinyl chloride (PVC) frame box (30 × 30 × 30 cm), (2) a fly entrance module, and (3) a black fly net (30 × 30 × 30 cm) [16]. Each trap was baited with 250 g of 1-day-old beef tainted by leaving at room temperature for 24 hours [17]. The bait was kept in a translucent plastic container and placed underneath the fly entrance module for the duration of trapping. After the three-hour collection period, the fly net was sealed, detached from the PVC frame, individually placed in transparent plastic bags, and transported to the laboratory at the College of Medicine and Public Health, Ubon Ratchathani University. At the laboratory, flies were sacrificed by placing the plastic bags from each trap into a freezer set at −20°C for 15 min. All flies were counted, sexed, and identified the lowest taxonomic resolution possible using the taxonomic keys from Tumrasvin et al. [18] and Kurahashi and Chowanadisai [19].

### 2.2. Statistical Analysis

Following identification and abundance calculations for each site and species a chi-square test was used to compare between the two predominant species,* C. megacephala* and *M. domestica,* from the five collection sites. A Kruskal-Wallis Analysis of Variance (KW-ANOVA) followed by individual Mann-Whitney *U* tests were used to test for statistical differences among sites between the two predominant species at each site in each district. To analyze the abundance of *C*. *megacephala* and *M*. *domestica* over time, the data were first transformed with the natural log (ln(*X* + 1)) to normalize the data. An analysis of variance (ANOVA) was then conducted for each species using the Univariate General Linear Model (GLM) in SPSS 16.0 (SPSS Graduate Pack for Windows 16.0.1, Chicago, IL, USA). The model consisted of the variables location (Muang Ubon Ratchathani or Warinchamrap), month of collection and the interaction between location and month. Significance for all tests was observed at the *α* = 0.05 level.

## 3. Results

During the six months sampled, a total of 3,262 flies were collected from the ten collection sites. The surveys in Muang Ubon Ratchathani and Warinchamrap districts yielded a total of 2,257 and 1,005 flies, respectively (Tables 1 and 2). Species from all three main synanthropic fly families of Muscidae, Calliphoridae, and Sarcophagidae were recovered from the surveys. The dominant species captured was the blow fly, *C. megacephala*, which ranked first in prevalence at all the collection sites, followed by *M. domestica*.

**Figure 2:**
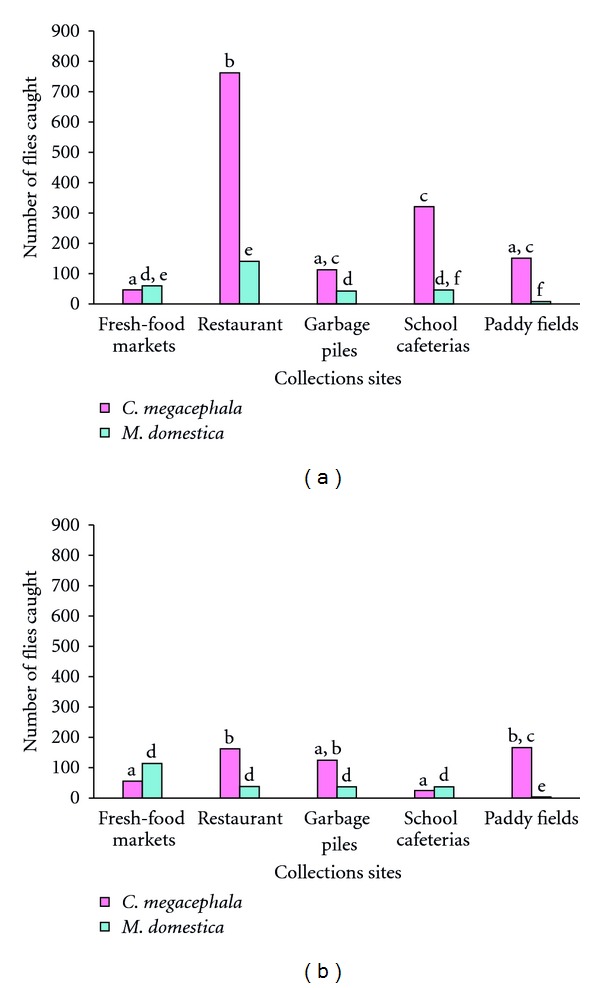
The abundance of the two predominant species, *C. megacephala* and *M. domestica* collected from various human habitations in Muang Ubon Ratchathani (a) and Warinchamrap (b), Ubon Ratchathani province, Northeast Thailand during September 2010–February 2011. Non-overlapping letters indicate a statistically significant difference in abundance among sites as determined by Kruskal-Wallis ANOVA and Mann-Whitney *U* comparisons for each species in each district at the *α* = 0.05 level.

At the five collection sites in Muang Ubon Ratchathani, six species of blow flies were found with* C. megacephala* as the most prevalent at 61.76% followed by *Achoetandrus rufifacies*, *Hemipyrellia ligurriens*, *Lucilia cuprina*, *Ceylonomyia nigripes,* and *Hemipyrellia pulchra, *subsequently. Within the Muscidae,* M. domestica* was most abundant at 13.20% and *Boettcherisca peregrina* was the dominant species of Sarcophagidae at 0.53% of the total flies collected (Table 1). The highest abundance of synanthropic flies were recovered from the restaurant sites at 46.57% followed by school cafeterias, paddy fields, garbage piles, and fresh-food markets at 23.79%, 16.54%, 7.74%, and 5.17% of total catch, respectively. The highest number of *C. megacephala* was recorded in Muang Ubon Ratchathani from the restaurant sites, whereas the lowest number was collected at the fresh-food market sites (Table 1 and Figure 2(a)). *M. domestica* was also most abundant at the restaurant site, but it was least abundant at the paddy field (Table 1 and Figure 2(a)). Collections of *C. nigripes* and* H. pulchra* were made only at the paddy field sites. Eleven species of flesh flies were recorded and scattered among all the collection sites (Table 1). The number of females collected was greater than males at all collection sites, with female to male ratio at 3.2 : 1, 4.6 : 1, 4.6 : 1, 5.2 : 1, and 7.6 : 1 in restaurant, school cafeterias, paddy fields, fresh-food markets, and garbage piles, respectively (Table 1).

In Warinchamrap, blow flies were numerically dominant with* C. megacephala* being the primary species collected (51.44%). These observations differ with respect to the relative abundances of blow flies collected from the different sites when compared to those recorded in Muang Ubon Ratchathani.* H. ligurriens* was the second most abundant species followed by *A. rufifacies*, *L. cuprina*, and *H. pulchra*, subsequently. No *C. nigripes* were collected from Warinchamrap during this collection period. *M. domestica* ranked first among collected flies in Muscidae (22.19%); while *B. peregrina* was the dominant species of flesh fly at 0.50% of the total number collected (Table 2). The garbage piles showed the highest abundance of synanthropic flies collected from Warinchamrap (39.00%), followed by restaurant, paddy field, fresh-food market, and school cafeteria sites, respectively. The highest number of *C. megacephala* recorded in this district was at the garbage piles, whereas the lowest number was found at the fresh-food markets (Table 2 and Figure 2(b)). *M. domestica* was the most abundant at the fresh-food markets, but the lowest abundance was at the paddy fields (Table 2 and Figure 2(b)). Six species of flesh flies were collected among all the sites. Similar to the results observed in the Muang district, the number of females collected exceeded males, with the female:male ratio being 3.5 : 1, 4.6 : 1, 5.2 : 1, and 7.9 : 1 in the paddy fields, restaurant, fresh-food markets, and school cafeterias, respectively. In contrast to the Muang district, flies collected from garbage piles indicated fewer females than males, with a female:male ratio of 0.65 : 1 (Table 2). 

Fly abundance varied across collection site type. A comparison of these two species among collection sites shows that significantly more *C*. *megacephala* were collected at the restaurant sites than any other type of site in the Muang Ubon Ratchathani (Figure 2(a)). The pattern for *M*. *domestica* is similar with significantly more flies collected at restaurants than at any other site except for fresh-food markets (Figure 2(a)). Fewer flies were collected at all sites in the Warinchamrap district overall with significantly more *C*. *megacephala* collected in the restaurant and paddy fields sites and significantly fewer *M*. *domestica* collected in the paddy fields (Figure 2(b)).

**Figure 3:**
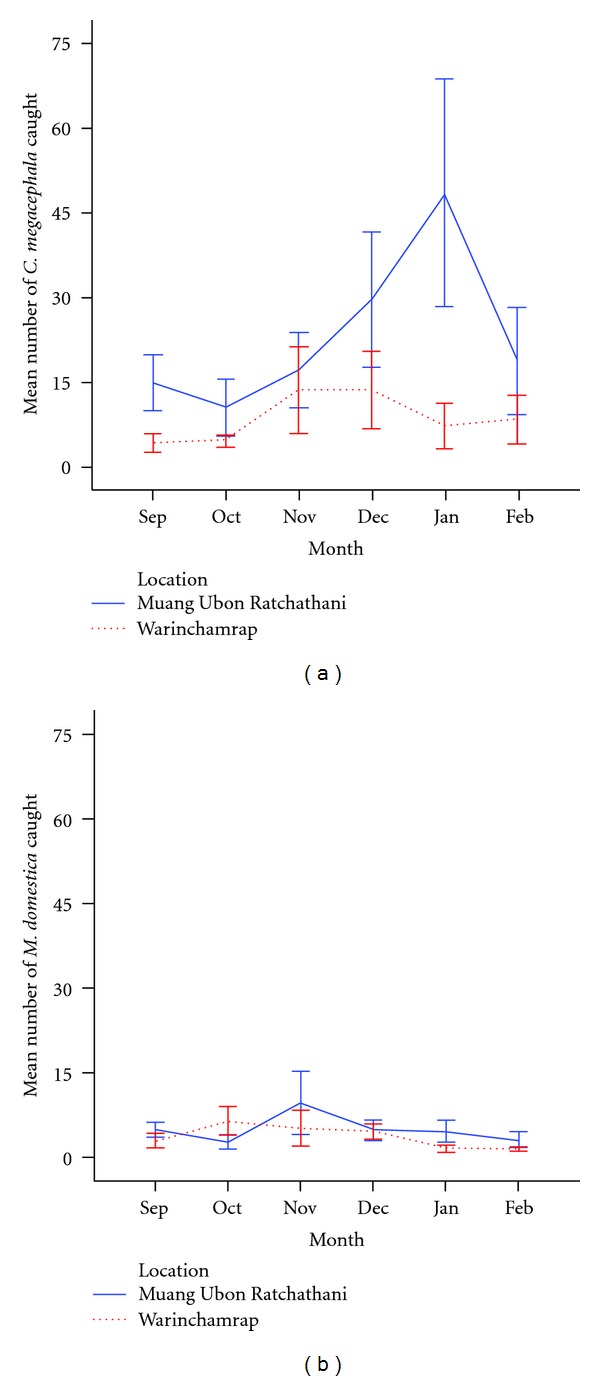
Mean abundance (+/− SE) of *C. megacephala* (a) and *M. domestica* (b) over September 2010–February 2011 from each location in Ubon Ratchathani province, Northeast Thailand.

A significant difference was observed between the prevalence of the two dominant species, *C. megacephala* and *M. domestica*, collected from Muang Ubon Ratchathani and Warinchamrap at all collection sites (*P* < 0.001) (Table 3). Comparing the two districts over time illustrates these differences, suggesting seasonality to *C*. *megacephala* populations in the Muang Ubon Ratchathani, but not in Warinchamrap (Figure 3(a)). The ANOVA results showed a statistically significant difference between locations (*F*_1,108_ = 11.493; *P* = 0.001) with significantly more *C*. *megacephala* collected in Muang Ubon Ratchathani. There was not a significant difference among months (*F*_5,108_ = 0.670; *P* = 0.647) or in the interaction between location and month (*F*_5,108_ = 0.324; *P* = 0.898). This pattern was not clearly defined in *M*. *domestica* (Figure 3(b)) and there was no significant difference in the mean abundance of house flies collected between the two sites (*F*_1,108_ = 0.846; *P* = 0.360). There were also no significant differences found among months (*F*_5,108_ = 0.754; *P* = 0.585) or in the interaction between location and month (*F*_5,108_ = 1.132; *P* = 0.348).

## 4. Discussion

The fly species collected in this survey represented 20 species in three families of Diptera (Muscidae, Calliphoridae, and Sarcophagidae). *C. megacephala* was the dominant species in paddy field, restaurant, and garbage pile sites, which is similar to the results of previous surveys of synanthropic flies in urban, suburban, forest, and mountain areas of Chiang Mai province in Northern Thailand [16]. On the other hand, previous surveys in Northern, Northeastern, and Central Thailand have suggested that *M. domestica* was the most abundant species in all the of the areas surveyed by Sucharit and Tumrasvin [15]. The numerical dominance of *M*. *domestica* was also observed in previous studies in Malaysia [11, 12] and southern England [10]. It is interesting to note that *M. domestica* was not the dominant species collecting in this study, although the collection sites were considered suitable breeding places for this species, which has strong associations with urban areas throughout the world [4]. One reason for the differences between the current study and others conducted in similar types of habitats, may be the use of only 1-day tainted beef as bait in the present study. Nurita et al. [11] suggested that adult blow flies are more attracted to carrion and soggy, bloody or soiled hair, fur, or wool; using these resources as protein sources for egg maturation as well as for egg-laying substrate [20]. The strong association of house flies with fecal material and wet waste may suggest that muscid flies may not be as strongly attracted to a carrion based bait thus affecting collection numbers observed in this study. However, previous studies in Thailand have indicated that 1-day tainted beef viscera was the most suitable bait to use for collecting both blow fly and house fly in the field [16]. The species of flesh flies collected in low numbers in this study may indicate a lack of attraction to this particular type of bait or a lower population abundance in these habitats.

This study revealed that more females were collected than males at almost all the study sites, with the exception of garbage piles in Warinchamrap district. This phenomenon was similar to previous investigations in Thailand [14–16] and Malaysia for both Muscidae and Calliphoridae [12], in the Czech Republic for Calliphoridae [21] and in Argentina for Muscidae [21]. This sexual asymmetry may be attributed to the fact that baits provide females with proteins needed for ovary development and as potential oviposition sites. However, the present results differ from a previous study in Thailand [15], reporting a 1 : 1 ratio of female:male in garbage piles among species in Muscidae, Callipholidae, and Sarcophagidae.

In the present study conducted in Muang Ubon Ratchathani and Warinchamrap, the dominant species was *C. megacephala* except at the school cafeteria sites in the Warinchamrap district, where *M. domestica* was dominant. The significantly high abundance of *C*. *megacephala* at restaurant sites in the Muang Ubon Ratchathani suggests an attractive resource at these sites. Bunchu et al. [17] found male and female *C*. *megacephala* were attracted to a wide variety of animal meat products as well as fresh fruits which are likely to be found in restaurants during food preparation. Sucharit and Tumrasvin [15] reported that *C. megacephala *collected from the Northeastern part of Thailand were found at garbage piles more than in market places, which is similar to the trend observed in this study. It is possible that the garbage piles are more attractive to *C. megacephala* because this location is composed of a variety of decomposing materials, such as vegetables and other decaying organic matter. On the other hand, such types of decaying organic matter are probably less abundant in fresh-food markets and school cafeterias, potentially explaining the lower number of *C. megacephala* captured at these sites. Such information may suggest that sanitation can have a significant impact on the availability of food and breeding places for synanthropic flies, thus affecting fly population densities in that particular human habitation. Poor sanitation practices may, therefore, increase the potential interaction between flies, enteropathogenic bacteria, and people.

In conclusion, this study illustrates the diversity of synanthropic flies collected in various human localities in Ubon Ratchathani province of Northeast Thailand. Few long-term fly survey datasets exist in Thailand and the data presented here suggest seasonality in *C*. *megacephala* abundance (Figure 3(a)), however, differences among months were not significant for the survey dates analyzed in this study. Additional long-term data should be collected to determine if this trend is significant. Such information may help in the development of control programs and in developing education programs to emphasize the importance of sanitation for fly management in this particular area. This is also the first survey of the Sarcophagidae associated with human developments in Thailand. These data may also prove useful in the development of data on forensically relevant flies in this region of Thailand. Overall, these data begin the establishment of a database that can be used to investigate other aspects of the synanthropic fly species associated with human environments and their ability to transmit pathogens to humans for this region of Thailand.

## Figures and Tables

**Table 1:** Species composition of synanthropic fly collected from various human habitations in Muang Ubon Ratchathani district, Ubon Ratchathani province, Northeast Thailand during September 2010–February 2011.

Number flies collected																			
Species	Fresh-food markets			Restaurant			Garbage piles			School cafeterias			Paddy fields			Total		Total	%
	F	M	Total	F	M	Total	F	M	Total	F	M	Total	F	M	Total	F	M
Family Calliphoridae																			
*Chrysomya megacephala*	37	10	47	554	208	762	105	8	113	247	74	321	139	12	151	1,082	312	1,394	61.76
*Achoetandrus rufifacies*	—	—	—	53	12	65	2	2	4	41	5	46	96	13	109	192	32	224	9.92
*Hemipyrellia ligurriens*	—	—	—	8	2	10	1	1	2	20	5	25	27	19	46	56	27	83	3.68
*Lucillia cuprina*	3	—	3	3	1	4	1	1	2	2	2	4	2	—	2	11	4	15	0.66
*Ceylonomyia nigripes*	—	—	—	—	—	—	—	—	—	—	—	—	2	—	2	2	—	2	0.09
*Hemipyrellia pulchra*	—	—	—	—	—	—	—	—	—	—	—	—	1	—	1	1	—	1	0.04
Other species	—	—	—	—	—	—	—	—	—	—	1	1	—	—	—	—	1	1	0.04

Family Muscidae																			
*Musca domestica*	51	9	60	121	20	141	37	6	43	43	3	46	6	2	8	258	40	298	13.20
*Hydrotaea* spp.	—	—	—	4	1	5	—	—	—	14	1	15	—	—	—	18	2	20	0.88
*Atherigona* spp.	—	—	—	6	—	6	—	—	—	8	—	8	—	—	—	14	—	14	0.62
*Hydrotaea spinigera*	—	—	—	3	2	5	—	—	—	—	—	—	—	—	—	3	2	5	0.22
*Hydrotaea chalogaster*	—	—	—	—	—	—	1	—	1	—	—	—	—	—	—	1	—	1	0.04
*Dichaetomyia* spp.	—	—	—	—	—	—	—	—	—	—	1	1	—	—	—	—	1	1	0.04
Other Muscidae	—	—	—	29	5	34	—	—	—	47	—	47	—	—	—	76	5	81	3.59

Family Sarcophagidae																			
*Boettcherisca peregrina*	—	—	—	—	—	—	—	—	—	—	2	2	—	10	10	—	12	12	0.53
*Liopygia ruficornis*	—	—	—	3	—	3	—	—	—	1	1	2	—	—	—	4	1	5	0.22
*Parasarcophaga dux*	—	—	—	—	—	—	—	2	2	—	2	2	—	—	—	—	4	4	0.18
*Harpagophalla kempi*	—	—	—	—	—	—	—	—	—	—	—	—	—	3	3	—	3	3	0.13
*Parasarcophaga brevicornis*	—	—	—	—	—	—	—	—	—	—	1	1	—	1	1	—	2	2	0.09
*Parasarcophaga scopariiformis*	—	—	—	—	—	—	—	—	—	—	—	—	—	2	2	—	2	2	0.09
*Boettcherisca nathani*	—	—	—	—	—	—	—	—	—	—	—	—	—	1	1	—	1	1	0.04
*Myorhiana caudagalli*	—	—	—	—	—	—	—	—	—	—	—	—	—	1	1	—	1	1	0.04
*Parasarcophaga albiceps*	—	—	—	—	1	1	—	—	—	—	—	—	—	—	—	—	1	1	0.04
*Sinonipponia komi*	—	—	—	—	—	—	—	—	—	—	—	—	—	1	1	—	1	1	0.04
*Sinonipponia harinasuitai*	—	—	—	—	—	—	—	—	—	—	—	—	—	2	2	—	2	2	0.09
*Sarcophaga* spp.	7	—	7	17	—	17	8	—	8	17	—	17	34	—	34	83	—	83	3.68

Total (%)	98	19	117 (5.18)	801	252	1,053 (46.65)	155	20	175 (7.75)	440	98	538 (23.84)	307	67	374 (16.50)	1,801	456	2,257 (100)	100

F: Female, M: Male.

**Table 2:** Species composition of synanthropic fly collected from various human habitations in Warinchamrap district, Ubon Ratchathani province, Northeast Thailand during September 2010–February 2011.

Number flies collected																			
Species	Fresh-food markets			Restaurant			Garbage piles			School cafeterias			Paddy fields			Total		Total of flies caught	%
	F	M	Total	F	M	Total	F	M	Total	F	M	Total	F	M	Total	F	M
Family Calliphoridae																			
*Chrysomya megacephala*	45	9	54	141	16	157	23	98	242	24	—	24	125	36	161	358	159	517	51.44
*Hemipyrellia ligurriens*	4	—	4	24	13	37	5	—	10	4	—	4	28	10	38	65	23	88	8.76
*Achoetandrus rufifacies*	—	—	—	1	—	1	18	5	46	—	—	—	15	—	15	34	5	39	3.88
*Lucillia cuprina*	5	—	5	8	2	10	1	1	4	—	—	—	—	1	1	14	4	18	1.79
*Hemipyrellia pulchra*	—	—	—	1	—	1	—	—	—	—	—	—	8	2	10	9	2	11	1.09
Other Callipholidae	—	—	—	—	—	—	—	—	—	—	—	—	2	—	2	2	—	2	0.20

Family Muscidae																			
*Musca domestica*	85	25	110	24	13	37	21	15	72	28	8	36	4	—	4	162	61	223	22.19
*Atherigona* spp.	39	—	39	5	—	5	—	—	—	—	—	—	—	—	—	44	—	44	4.38
*Hydrotaea* spp.	4	—	4	4	—	4	—	—	—	—	—	—	—	—	—	8	—	8	0.80
*Dichaetomyia* spp.	—	—	—	—	—	—	—	—	—	—	—	—	1	—	1	1	—	1	0.10
Other Muscidae	—	1	1	—	—	—	—	—	—	—	—	—	—	—	—	—	1	1	0.10

Family Sarcophagidae																			
*Boettcherisca peregrina*	—	—	—	—	2	2	—	—	—	—	—	—	—	3	3	—	5	5	0.50
*Liopygia ruficornis*	—	—	—	1	—	1	2	—	4	—	—	—	—	—	—	3	—	3	0.30
*Sarcorohdendrofia princeps*	—	—	—	—	—	—	—	—	—	—	—	—	—	3	3	—	3	3	0.30
*Harpagophalla kempi*	—	1	1	—	1	1	—	—	—	—	—	—	—	—	—	—	2	2	0.20
*Parasarcophaga brevicornis*	—	—	—	—	—	—	—	—	—	—	—	—	—	1	1	—	1	1	0.10
*Parasarcophaga dux*	—	—	—	—	1	1	—	—	—	—	—	—	—	—	—	—	1	1	0.10
*Sarcophaga* spp.	5	—	5	9	—	9	7	—	14	7	—	7	10	—	10	38	—	38	3.78

Total (%)	187	36	223 (22.19)	218	48	266 (26.47)	77	119	392 (39.00)	63	8	71 (7.06)	193	56	249 (24.78)	738	267	1,005 (100)	100

F: Female, M: Male.

**Table 3:** Comparison between the two predominant fly species,* C. megacephala* and *M. domestica* from five collection sites in Ubon Ratchathani province, Northeast Thailand during September 2010–February 2011.

Species	Collection sites					Chi-square (*P*-value)
	Fresh-food markets	Restaurant	Garbage piles	School cafeterias	Paddy fields
*M. domestica*	110 (67.0%)	37 (19.1%)	72 (23.0%)	36 (60.0%)	4 (2.5%)	216.2 (<0.001)
*C. megacephala*	54 (33.0%)	157 (80.9%)	242 (77.0%)	24 (40.0%)	161 (97.5%)	
